# Uterine *Pgrmc2* Deficiency Attenuates Endometrial Hyperplasia and Cancer and Prolongs Lifespan in a *Pten* Loss-of-Function-Induced Cancer Model

**DOI:** 10.3390/cancers17071178

**Published:** 2025-03-31

**Authors:** Nicole C. Kelp, Cindy A. Pru, Sandeep Paudel, John P. Lydon, J. Julie Kim, John J. Peluso, James K. Pru

**Affiliations:** 1 Center for Reproductive Biology, School of Molecular Biosciences and Department of Animal Sciences, Washington State University, Pullman, WA 99163, USA; nicole.kelp@colostate.edu (N.C.K.);; 2 Department of Microbiology, Immunology, and Pathology, Colorado State University, Fort Collins, CO 80523, USA; 3 Program in Reproductive Biology, Department of Animal Science, University of Wyoming, Laramie, WY 82071, USA; sandeep.bchem@gmail.com; 4 Department of Molecular and Cellular Biology, Baylor College of Medicine, Houston, TX 77030, USA; jlydon@bcm.edu; 5 Division of Reproductive Science in Medicine, Department of Obstetrics and Gynecology, Northwestern University, Chicago, IL 60611, USA; j-kim4@northwestern.edu; 6 Departments of Cell Biology and Obstetrics and Gynecology, University of Connecticut Health Center, Farmington, CT 06030, USA; peluso@uchc.edu

**Keywords:** cancer, endometrial, hyperplasia, PGRMC1, PGRMC2, progesterone, PTEN, uterus

## Abstract

The expression of members of the progesterone receptor membrane component (PGRMC) family, particularly PGRMC1, is elevated in diverse types of cancers, particularly those of the female reproductive system. While xenograft tumor studies using human transformed cell lines in immunocompromised mice have suggested that *PGRMC1* enhances tumor growth and chemoresistance, the exact role of members of the PGRMC family in cancer development in vivo remains unclear. In this study, we examined the effect of deleting *Pgrmc2* on the development of endometrial hyperplasia and cancer using a murine *phosphatase and tensin homologue* (*Pten*) conditional loss-of-function model. We previously established that PGRMCs are cell survival factors that are required for normal estrogen-induced uterine epithelial cell proliferation and normal female fertility. The deletion of *Pgrmc2* reduced the incidence and severity of endometrial hyperplasia and cancer in mice with conditional *Pten*-heterozygous uteri and increased lifespan in mice with conditional *Pten*-knockout uteri. Mechanistically, the deletion of *Pgrmc2* decreased uterine glandular epithelial cell proliferation. *Pten* loss-of-function-induced endometrial hyperplasia and cancer elevated uterine inflammation, but this was not impacted by PGRMC2 deficiency. This study identifies PGRMC2 as a potential therapeutic target to be inhibited in the treatment of endometrial hyperplasia and cancer, particularly where PTEN activity is reduced due to gene mutation or loss.

## Simple Summary

The tumor suppressor *phosphatase and tensin homologue* (*PTEN*) is the most frequently mutated or deleted gene in endometrioid endometrial carcinoma (i.e., usually Type I endometrial cancer). The conditional mutagenesis of *Pten* alleles in the murine female reproductive tract recapitulates the development of endometrial hyperplasia and cancer with a similar consistency found in women. Identifying targets to reduce the incidence and severity of *Pten* loss-of-function-induced endometrial hyperplasia and cancer is critical for developing better treatment options for this disease. Using a murine model, the ablation of *progesterone receptor membrane component 2* (*Pgrmc2*) was shown to attenuate the timing, aggression, and lethality of *Pten* loss-of-function-induced endometrial hyperplasia and cancer.

## 1. Introduction

Endometrial cancer is the fourth most common cancer in women in the United States, and the most common gynecologic cancer. Based on estimates from the American Cancer Society, 67,880 women were diagnosed with endometrial cancer in 2024 in the U.S., and an estimated 13,250 died from the disease [1]. While hysterectomy is the leading treatment option, better therapies and earlier detection methods are needed to increase survival rates, particularly in younger women hoping to have children. In endometrioid endometrial carcinoma (EEC), 17-β-estradiol (E2) drives epithelial cell proliferation while progesterone (P4) inhibits E2-induced epithelial cell proliferation. Thus, the unopposed actions of E2 can lead to endometrial carcinoma, and P4 analogs are often used as a treatment option [2]. E2 signaling indirectly activates the AKT pathway of cell survival and proliferation. A negative regulator of AKT signaling is the phosphatase and tensin homologue (PTEN). The loss of PTEN activity via gene mutation leads to elevated AKT activity and increased endometrial epithelial cell proliferation [3]. Inflammation likely plays a prominent role in the development and progression of endometrial cancer [4]. Notably, PTEN is mutated in 83% of type I endometrial carcinomas [5]. In mice, the deletion of both alleles of *Pten* in the uterus leads to endometrial cancer in 100% by one month of age, and *Pten* heterozygosity results in 100% hyperplasia and 22% endometrial cancer [6,7,8]. *Pten* heterozygosity in mice is often used as a model to study endometrial oncogenesis in an effort to assess the contributions of other genes thought to be involved in endometrial neoplasm formation or those that prevent the development and progression of the disease (e.g., [9,10,11]).

Progesterone receptor membrane component (PGRMC) family members PGRMC1 and PGRMC2 were first cloned from porcine vascular smooth muscle cells [12] and human cells [13]. Whereas PGRMC1 expression is highest in the liver and kidney, PGRMC2 expression is most abundant in placenta [13]. Both proteins are expressed in most tissues. Initial characterization studies using porcine liver fractions demonstrated that PGRMC1 binds steroid hormones with affinities in the low- to mid-nanomolar range [14]. PGRMC1 was found to have the highest affinity for P4 (i.e., 11 nM), but it also bound corticosterone, cortisol, testosterone, and cholesterol. PGRMC1 has a poor affinity for 17-β estradiol and aldosterone [14]. Despite being labelled as a P4 receptor, PGRMC2 has not yet been shown to bind and become activated by P4. PGRMC proteins have pleiotropic functions in female reproduction and P4 signaling [15,16,17], mitosis and meiosis [18], energy metabolism and Warburg glycolysis [19], angiogenesis [20], receptor trafficking to the plasma membrane [21], drug metabolism [22], heme transport [23], and neural development [24]. Gene mutations and aberrant expression profiles in PGRMC1 and PGRMC2 are associated with a host of reproductive diseases [15,25,26]. Through association-based studies in humans, as well as cell culture and xenograft studies in immunocompromised mice, PGRMC1 is thought to contribute to the development and progression of solid tumors in many different organs [26,27,28,29,30,31,32]. Similarly, much of what is known about PGRMC2 stems from descriptive studies in cell lines and reproductive tissues [15,25,26,27]. PGRMC2 expression correlates with the development of endometriosis in a non-human primate model of the disease (reviewed in [26]). The conditional ablation of *Pgrmc2* in mice results in subfertility that progresses to premature reproductive senescence [16,26,27]. PGRMC2 was shown to function in adipocytes as an essential intracellular heme chaperone required for normal function [23], as well as a pressure–volume regulator for accommodating stress responses in cardiomyocytes [33]. PGRMC2 plays an important role in human placental extravillous trophoblast invasion during early pregnancy where it also helps regulate immune homeostasis at the maternal–fetal interface [34,35]. Considerably less is known about a role for PGRMC2 in cancer. As with PGRMC1, PGRMC2 is elevated in ovarian cancer and a large number of ovarian cancer cell lines [27]. However, to date, we are unaware of any studies that assess the function of PGRMC2 in the context of the development or progression of cancer in vivo.

Given that PGRMC proteins are generally overexpressed in many tumor types, we hypothesize that selectively deleting a PGRMC family member from the endometrium with the heterozygous or complete loss of *Pten* would prevent the development of or reduce the severity of *Pten* loss-of-function-induced hyperplasia and cancer. Because of the general lack of information about PGRMC2 in the context of cancer, *Pgrmc2* was specifically evaluated in this context.

## 2. Materials and Methods

### 2.1. Animals

All animal protocols were approved by the Institutional Animal Care and Use Committees at Washington State University or the University of Wyoming. The floxed *Pgrmc2* mice (*Pgrmc2^fl^*) and their use with *Pgr^cre/+^* mice were described previously [16]. Floxed *Pten* (*Pten^fl/fl^*) mice were obtained from Jackson laboratories. Mice of 10 different genotypes were generated for this study by crossing *Pgr^cre/+^* mice with *Pten^fl/fl^* and/or *Pgrmc2^fl/fl^* mice. Tissues were collected from mice at 6–12 weeks of age or at 9 months of age at the completion of the study. In some cases, such as with *Pgr^cre/+^*; *Pten^fl/fl^* (*Pten^d/d^*) mice, animals were euthanized at 4–9 months of age due to rapid development of cancer. Animals aged 4–9 months were first ovariectomized and tissues were collected 1 week later for fixation in 4% paraformaldehyde (PFA) and paraffin embedded or snap frozen in liquid nitrogen and stored at −80 °C until use. For assessing the endometrial epithelial mitotic response to 17β-estradiol (E2), young (6–12 weeks) *Pten^+/fl^*; *Pgrmc2^fl/fl^*, *Pten^+/d^*, and *Pten^+/d^; Pgrmc2^d/d^* mice were ovariectomized, allowed to rest for 1–2 weeks, and then subjected to sesame oil vehicle treatment that mirrored treatment with E2 (100 ng E2 for 2 days, 5 days without treatment, 50 ng E2, and tissue collection 18 h later). Tissues were again collected for paraffin embedding and some were snap frozen.

### 2.2. Histology and Immunohistochemistry

All human and mouse tissues were fixed in 10% buffered formalin or PFA, then stored in 70% ethanol until paraffin embedding. Tissues were processed through a graded series of ethanol (70–100%) and xylenes, embedded in paraffin, and sectioned at 5 µm by microtome. Human tissues were collected deidentified, and contributed to a Northwestern University tissue repository as part of NCATS/UH3TR00120. Proliferative and secretory specimens were obtained from women with benign gynecological conditions such as fibroids. For general histological analyses, tissue sections were deparaffinized with xylenes, rehydrated, and stained with hematoxylin and eosin. Immunohistochemistry (IHC) was completed as previously described [16,17,31,32]. Antibodies used for IHC included anti-phosphohistone H3 (phH3) primary antibody (Ser10, Millipore 06-570, 1:750, Burlington, MA, USA), anti-PGRMC2 primary antibody (Sigma, 1:100, St. Louis, MO, USA), and biotinylated anti-rabbit IgG secondary antibody (Vector BA-1000, 1:750, Burlingame, CA, USA). From young ovariectomized *Pten^+/fl^*; *Pgrmc2^fl/fl^*, *Pten^+/d^*, and *Pten^+/d^; Pgrmc2^d/d^* mice treated with vehicle or E2 and aged ovariectomized *Pten^+/fl^*; *Pgrmc2^fl/fl^*, *Pgrmc2^d/d^*, *Pten^+/d^*, and *Pten^+/d^; Pgrmc2^d/d^* mice, mitotic (phH3+ cells) counts were made from three different tissue sections for each biological replicate. From 9-month-old *Pten^+/fl^; Pgrmc2^fl/fl^*, *Pten^+/d^*, and *Pten^+/d^; Pgrmc2^d/d^* mice ovariectomized one week prior to euthanasia, the number of granulated and degranulated mast cells was counted on a per section basis. Following deparaffinization and rehydration, 5 µm sections were stained with toluidine blue working solution (1 mg/mL) and counter-stained with hematoxylin. Mast cells were quantified by counting dark blue cells and establishing a mean value from three non-adjacent sections for each biological replicate.

### 2.3. RNA Isolation and qPCR

For assessing mRNA levels of the classical estrogen receptor (*Esr1*) and progesterone receptor (*Pgr*) from uterine tissues collected from ovarictomized and sesame oil vehicle treated *Pten^+/fl^*; *Pgrmc2^fl/fl^*, *Pten^+/d^*, and *Pten^+/d^; Pgrmc2^d/d^* mice, total cellular RNA was isolated using TRI-Reagent (Sigma, St. Louis, MO, USA). RNA samples were subjected to DNAse I digestion (Promega, Madison, WI, USA), and complementary DNA was generated using iScript Reverse-Transcription Supermix (BioRad, Hong Kong, China). qPCR was performed to compare expression of *Esr1* and *Pgr* in complementary DNA samples. *Rpl13a* was included for normalization. A negative control (no reverse transcription) was included to confirm the absence of genomic DNA. Table 1 provides primer information for qPCR, as well as for primers used for genotyping animals.

### 2.4. Data Analyses

Animals with the same genotype were randomly assigned to the various treatment groups. All data are presented as the mean ± SEM for n = 3–28 samples. Individual animals represented a single biological replicate. Differences between genotypes were assessed by Student’s *t*-test, where the mean values of two groups were compared. Differences between treatment groups or genotypes with more than two groups were analyzed by one-way analysis of variance followed by Tukey’s post hoc test. A chi-square analysis was used for incidence studies and a Kaplan–Meier survival curve was generated for lifespan analyses. For semi-quantitatively comparing PGRMC2 protein levels in human endometrial tissues, a composite h-score was established by multiplying the staining intensity [scored as 1 (low) or 2 (high)] and percentage of positive cells [scored as 0 (no staining), 1 (1–10% staining), 2 (11–50% staining), or 3 (>50% staining)] within each tissue specimen (n=10 each). Contingency graphs were then generated to show the relationship between staining intensity and the percentage of positive cells by showing the frequencies of different combinations of these variables. All data were analyzed using GraphPad 5.0 software (San Diego, CA, USA) where *p* ≤ 0.05 was considered statistically significant and *p* > 0.05 ≤ 0.10 was considered a trend.

## 3. Results

### 3.1. PGRMC2 Expression in Human Endometrium and Endometrial Carcinoma

**Figure 1:**
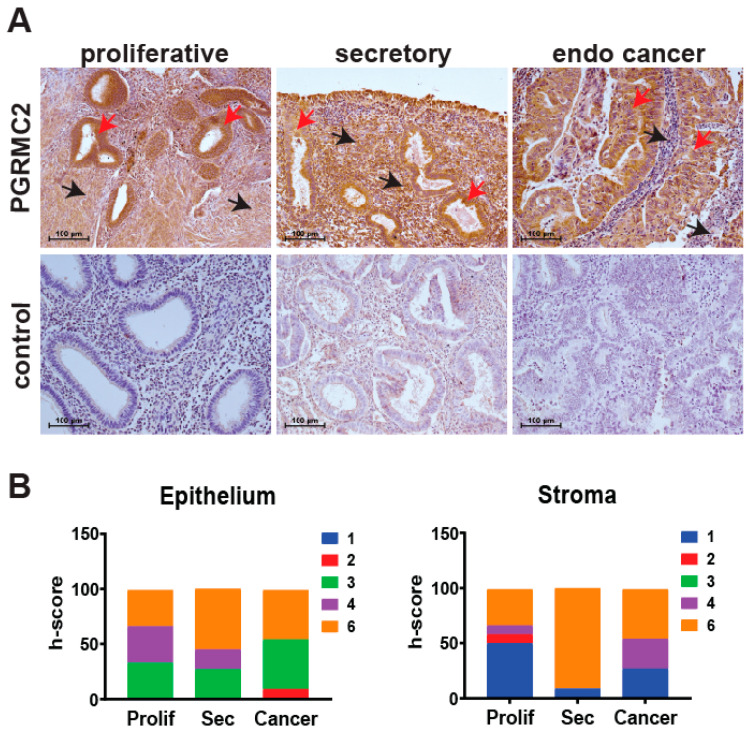
Human endometrial PGRMC2 protein levels during the menstrual cycle and in endometrial cancer. (**A**) Localization of PGRMC2 (brown stain) in human endometrial stroma (black arrows) and epithelia (red arrows) collected during the proliferative and secretory phases of the menstrual cycle and endometrial (endo) cancer detected by immunohistochemistry. Lower panels show sections stained without primary antibody as a negative control. (**B**) Contingency plots comparing semi-quantitative h-score values for PGRMC2 in endometrial epithelial and stromal tissues. The h-score index ranges from low (blue) to high (orange). N = 10, scale bar = 100 µm.

The level of PGRMC2 protein was evaluated by immunohistochemistry in human endometrial samples during the proliferative and secretory phases of the menstrual cycle, as well as in endometrial cancer specimens (Figure 1). Semi-quantitative h-scoring was used to show that PGRMC2 protein levels did not change in epithelial or stromal tissues across the different groups. During the proliferative phase, PGRMC2 was abundant in both the glandular and luminal epithelia and, to a lesser extent, in the stroma. PGRMC2 remained abundant in epithelia during the secretory phase, and appeared to be elevated in the stromal compartment compared with stromal tissue from the proliferative phase. However, a difference in PGRMC2 between epithelial and stromal tissues was not found to be statistically different during the menstrual cycle or endometrial cancer. PGRMC2 seemed more abundant in the neoplastic epithelium of endometrial cancer tissues when compared to adjacent stromal tissue, but this may likely be due to the presence of stratified epithelium in this neoplastic tissue.

### 3.2. Pgrmc2 Deficiency Reduces the Incidence and Severity of Pten Loss-of-Function-Induced Endometrial Hyperplasia and Carcinoma

**Figure 2:**
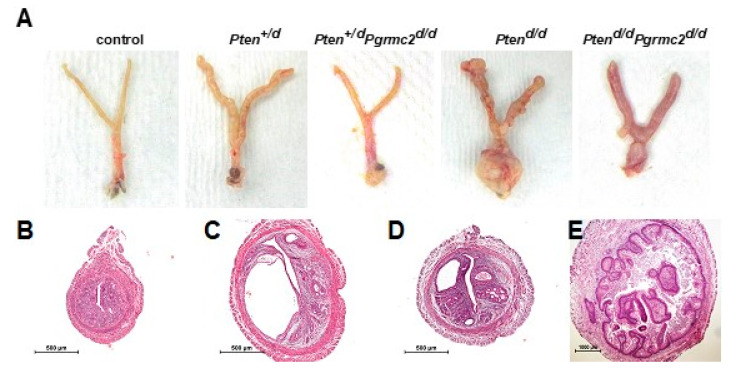
Gross anatomy of female reproductive tracts and uterine histology. (**A**) Gross reproductive tract anatomy from *Pten^+/fl^*; *Pgrmc2^fl/fl^* (control), *Pten^+/d^*, *Pten^+/d^*; *Pgrmc2^d/d^*, *Pten^d/d^*, *Pten^d/d^*, and *Pten^d/d^; Pgrmc2^d/d^* mice at one week after ovariectomy at 9 months of age. H&E-stained uterine cross-sections from (**B**) *Pten^+/fl^*; *Pgrmc2^fl/fl^* (control), (**C**) *Pgrmc2^d/d^*, (**D**) *Pten^+/d^; Pgrmc2^d/d^,* and (**E**) *Pten^d/^*^d^ mice. N = 3–28. scale bar = 100 µm.

The most commonly mutated gene in human endometrioid endometrial carcinoma is *PTEN* [36]. The deletion of a single *Pten* allele in mice results in a condition similar to Cowden’s syndrome in women. This phenotype is accompanied by endometrial hyperplasia in 100% of mice and endometrial carcinoma in 22% of mice by six months of age [37]. The conditional ablation of both alleles causes endometrial carcinoma in 100% of mice by around one month of age [6]. Conditional mutagenesis in mice in which one or both *Pten* alleles are ablated is commonly used as a model to study *Pten*-based endometrial carcinoma to understand how other gene mutations contribute to endometrial neoplasms [6,7,8,10,11,38]. Given the lack of in vivo information about a role for PGRMC2 in oncogenesis, we sought to determine if endometrial *Pgrmc2* ablation would impact the development and progression of endometrial neoplasia in a well-established model of endometrial cancer. In this study, the *Pgr^Cre/+^* mouse line was used in which Cre recombinase is expressed in cells of the female reproductive tract under the control of the *Pgr* promoter [39]. *Pgr^Cre/+^* mice were crossed with *Pten^fl/fl^* and/or *Pgrmc2^fl/fl^* mice to conditionally ablate one or both copies of these genes in *Pgr*-expressing cells. Figure 2A shows the gross morphology of female reproductive tracts isolated from control (*Pten^+/fl^*; *Pgrmc2^fl/fl^*), *Pten^+/d^*, *Pten^+/d^*; *Pgrmc2^d/d^, Pten^d/d^*, and *Pten^d/d^*; *Pgrmc2^d/d^* mice one week after ovariectomy at nine months of age. The exterior surface of tracts from *Pten^+/d^* and *Pten^d/d^* were rough compared to the smooth surface of tracts isolated from *Pten^+/d^; Pgrmc2^d/d^* and *Pten^d/d^*; *Pgrmc2^d/d^* mice. Overt signs of carcinoma were clearly present in *Pten^d/d^* mice, particularly at the distal end of the uterus near the oviduct and the cervix. Images of the H&E-stained uterine cross-sections are shown in Figure 2. No signs of hyperplasia or carcinoma were found in control mice (Figure 2B). As previously described [16], non-hyperplastic cystic glands formed in uteri from *Pgrmc2^d/d^* mice, consistent with a premature aging phenotype (Figure 2C). Also shown are representative images of atypical endometrial hyperplasia (Figure 2D) and carcinoma (Figure 2E) commonly observed in uteri isolated from *Pten^d/d^* and *Pten^d/d^*; *Pgrmc2^d/d^* mice.

**Figure 3:**
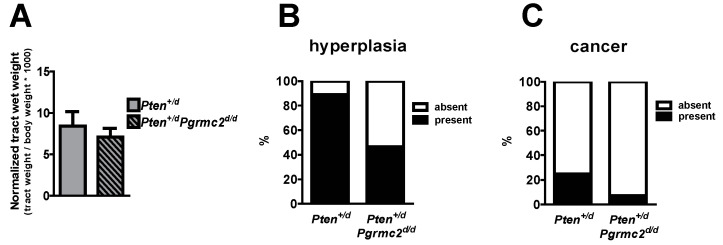
Endometrial hyperplasia and cancer in *Pten^+/d^* and *Pten^+/d^; Pgrmc2^d/d^* mice. Uterine wet weight (**A**), incidence of hyperplasia (**B**), and incidence of endometrial cancer (**C**) are compared between *Pten^+/d^* and *Pten^+/d^; Pgrmc2^d/d^* mice. n = 9 or 28.

**Figure 4:**
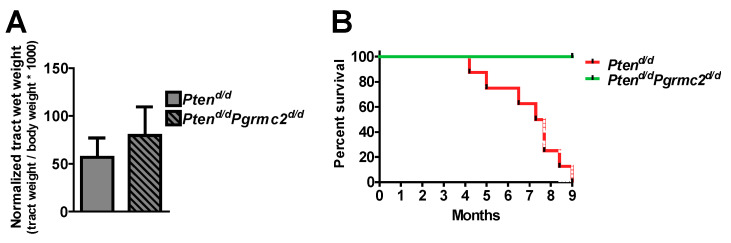
Impact of *Pgrmc2* ablation on *Pten* loss-of-function-induced cancer and lethality. (**A**) Comparison of normalized reproductive tract weight from *Pten^d/d^* and *Pten^d/d^Pgrmc2^d/d^* mice (*p* > 0.05). (**B**) Kaplan–Meier survival analysis comparing lethality of endometrial carcinoma in *Pten^d/d^* and *Pten^d/d^Pgrmc2^d/d^* mice. *p* ≤ 0.0001, n = 8–9.

The incidence of endometrial hyperplasia and cancer was determined in mice with 10 different *Pten* and *Pgrmc2* genotypes as outlined in Table 2. Reproductive tracts were collected for analyses at nine months of age, except for *Pten^d/d^* mice which were collected at 4–9 months of age due to the lethality of this phenotype. While the tract wet weight was not different between *Pten^+/d^* and *Pten^+/d^; Pgrmc2^d/d^* mice, hyperplasia was reduced from 88.9% in *Pten^+/d^* mice to 46.4% in *Pten^+/d^; Pgrmc2^d/d^* mice (Figure 3A,B). The ablation of *Pgrmc2* on a *Pten^+/d^* background also decreased the incidence of endometrial cancer to 7.1% compared to the 22.2% in *Pten^+/d^* mice (Figure 3C). Similarly, the tract weight did not differ between *Pten^d/d^,* and *Pten^d/d^; Pgrmc2^d/d^* mice (Figure 4). The ablation of *Pgrmc2* (i.e., *Pten^d/d^; Pgrmc2^d/d^*) did not reduce the 100% incidence of hyperplasia or cancer observed in *Pten^d/d^* mice (Table 2). However, whereas all *Pten^d/d^* mice had to be euthanized prior to the end of the study at nine months, 100% of *Pten^d/d^; Pgrmc2^d/d^* mice survived to nine months of age, suggesting that the ablation of *Pgrmc2* attenuated the progression of endometrial cancer (Figure 4B).

**Figure 5:**
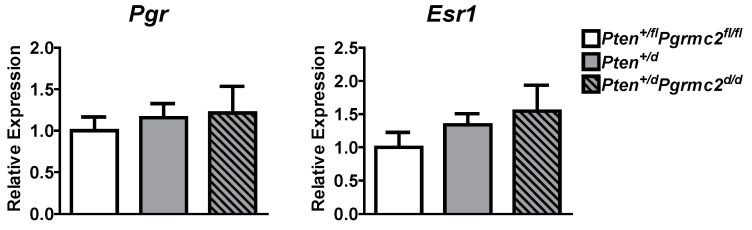
Messenger RNA levels of the classical progesterone receptor (*Pgr*) and estrogen receptor alpha (*Esr1*) were measured by qPCR in *Pten^+/fl^; Pgrmc2^fl/fl^*, *Pten^+/d^,* and *Pten^+/d^; Pgrmc2^d/d^* mice. n = 5.

**Figure 6:**
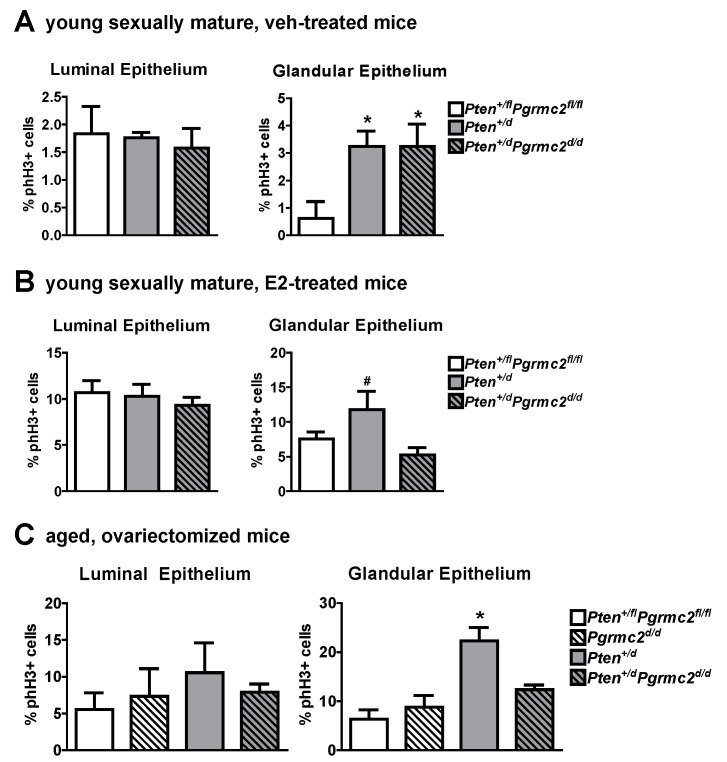
Effect of *Pten* heterozygosity and *Pgrmc2* ablation on endometrial epithelial cell mitosis in young and aged *Pten^+/fl^; Pgrmc2^fl/fl^*, *Pten^+/d^,* and *Pten^+/d^; Pgrmc2^d/d^* mice. The marker phospho-histone H3 (phH3) was used to quantify mitosis in endometrial luminal and glandular epithelia in young ovariectomized sexually mature *Pten^+/fl^; Pgrmc2^fl/fl^*, *Pten^+/d^*, and *Pten^+/d^; Pgrmc2^d/d^* mice treated with vehicle (veh, sesame oil) (**A**) or E2 (100 ng E2 for 2 days, 5 days without treatment, 50 ng E2, and tissue collection 18 h later) (**B**). (**C**) Quantification of basal mitosis in ovariectomized aged (i.e., 9 months) *Pten^+/fl^; Pgrmc2^fl/fl^*, *Pgrmc2^d/d^*, *Pten^+/d^,* and *Pten^+/d^; Pgrmc2^d/d^* mice. N = 3–4 per group, * *p* ≤ 0.05, ^#^ *p* = 0.07.

The mRNA levels of *Pgr* and *Esr1* were evaluated in uterine tissues from *Pten^+/fl^; Pgrmc2^fl/fl^* (control), *Pten^+/d^,* and *Pten^+/d^; Pgrmc2^d/d^* mice and found not to be different between the groups (Figure 5). Given that PGRMC proteins regulate proliferation in granulosa cells and xenograft tumors [15,18,25,26,27,30,32,40], we next evaluated endometrial luminal and glandular epithelial cell mitosis by phospho-histone H3 immunohistochemsitry in young sexually mature ovariectomized *Pten^+/fl^; Pgrmc2^fl/fl^*, *Pten^+/d^,* and *Pten^+/d^; Pgrmc2^d/d^* mice treated with either vehicle or E2. While there was no difference in basal luminal epithelial cell mitosis across genotypes, basal glandular epithelial mitosis was approximately six times higher in uterine tissue from *Pten^+/d^* and *Pten^+/d^; Pgrmc2^d/d^* mice than from *Pten^+/fl^; Pgrmc2^fl/fl^* mice. The ablation of *Pgrmc2* had no effect on the elevated basal mitosis observed in young *Pten^+/d^* mice (Figure 6A). The treatment of *Pten^+/fl^; Pgrmc2^fl/fl^* (control), *Pten^+/d^,* and *Pten^+/d^; Pgrmc2^d/d^* mice with E2 equitably elevated luminal epithelial cell proliferation to approximately 10%. The ablation of a single copy of the *Pten* gene tended (*p* = 0.07) to increase E2-induced glandular epithelial mitosis by about 40% over glandular epithelium from *Pten^+/fl^; Pgrmc2^fl/fl^* mice. Interestingly, the elevated mitosis observed in *Pten^+/d^* mice was lost upon the ablation of *Pgrmc2* in *Pten^+/d^; Pgrmc2^d/d^* mice (Figure 6B). In aged ovariectomized mice, basal endometrial luminal epithelial cell mitosis was not different in *Pten^+/fl^; Pgrmc2^fl/fl^*, *Pgrmc2^d/d^*, *Pten^+/d^,* and *Pten^+/d^; Pgrmc2^d/d^* mice. In contrast, glandular epithelial cell mitosis was significantly higher in *Pten^+/d^* mice than in all other groups, including *Pten^+/d^; Pgrmc2^d/d^* mice (Figure 6C).

**Figure 7:**
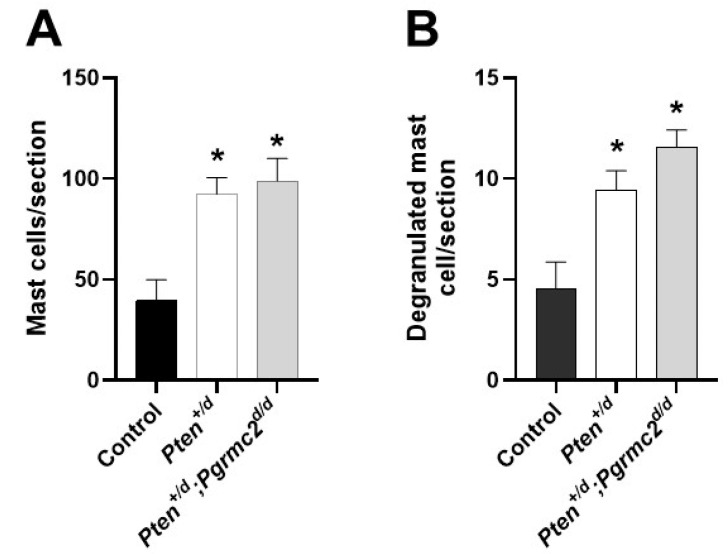
Uterine mast cell infiltration and degranulation in *Pten^+/fl^; Pgrmc2^fl/fl^*, *Pten^+/d^,* and *Pten^+/d^; Pgrmc2^d/d^* mice. The total number of toluidine-stained mast cells (**A**) and degranulated mast cells (**B**) was determined in uterine histological sections from 9-month-old ovariectomized *Pten^+/fl^; Pgrmc2^fl/fl^*, *Pten^+/d^,* and *Pten^+/d^; Pgrmc2^d/d^* mice. * *p* ≤ 0.05, n = 3.

Conditional *Pten* heterozygosity or deficiency in the uterus causes inflammation [6]. In the final study, we quantified the uterine infiltration of mast cells and their degranulation in *Pten^+/fl^; Pgrmc2^fl/fl^*, *Pten^+/d^,* and *Pten^+/d^; Pgrmc2^d/d^* mice. As shown in Figure 7, an approximate 2.5-fold increase in mast cells infiltrated occurred in uterine tissue from *Pten^+/d^* mice compared with *Pten^+/fl^; Pgrmc2^flfl^* control mice. The conditional ablation of *Pgrmc2* in *Pten^+/d^* mice failed to reduce mast cell infiltration. Approximately 10% of the total mast cells were degranulated in all tissues examined. As with total mast cells, the number of degranulated mast cells was about 2.5-fold higher in uterine tissues isolated from *Pten^+/d^* and *Pten^+/d^; Pgrmc2^d/d^* mice compared with uterine tissue from *Pten^+/fl^; Pgrmc2^fl/fl^* mice (Figure 7B).

## 4. Discussion

Coupled with supporting mutations in the cell cycle, adhesion, and/or apoptosis genes, mutations of tumor suppressor genes are causally responsible for neoplastic transformation in various organs. Endometrioid endometrial carcinoma, which generally exists as type 1 endometrial cancer, is the most common gynecological cancer. Mutations in the tumor suppressor gene *PTEN* occur in over 80% of type 1 endometrial cancer cases [5]. PTEN is a phosphatase that attenuates the activity of the AKT, a kinase activated by pro-survival and pro-growth ligand:receptor-initiated phosphorylation cascades. The loss of even one *PTEN* allele reduces the overall cellular PTEN phosphatase activity. This haploinsufficiency results in accelerated proliferation and neoplastic transformation in epithelial tissues of several organs due, in part, to its role in maintaining the chromatin structure and the integrity of the genome [3,41]. Because many tumor suppressor genes play vital roles in diverse normal cellular functions, the global ablation or mutation of these genes generally causes embryonic lethality. To circumvent this limitation, the *loxP-Cre* system allows for the ablation, mutation, or overexpression of genes in a cell-specific fashion [42]. The objective of this study was to conditionally ablate the *Pten* gene from endometrial cells and evaluate the consequences of concomitant *Pgrmc2* ablation. This was accomplished by using the *Pgr-Cre* mouse, which expresses Cre recombinase in cells that normally express the classical *Pgr*. Of note, an endometrial-specific Cre mouse line does not exist, so the *Pgr-Cre* mouse is routinely used to ablate, mutate, or overexpress genes in the endometrium despite having Cre recombinase expression in other organs of the female reproductive tract, peri-ovulatory follicles, mammary gland, and gonadotropes [39]. This approach has been used extensively to determine causal relationships between *PTEN* and other genes. For instance, deleting the *glucose-regulated protein-78* (*Grp78*) gene prevented the development of endometrial carcinoma in *Pten*-ablated mice [43]. Similarly, because *Pten* deletion deregulated lipid metabolism [7], the overexpression of an *mfat-1* transgene in mice reinstated lipid homeostasis and prevented *Pten*-heterozygous mice from developing hyperplasia and cancer [44]. The overexpression of the tumor suppressor *mitogen-inducible gene-6* (*Mig-6*) abrogated the development of *Pten*-loss-of function-induced endometrial carcinoma [45], whereas the ablation of *Mig-6* accelerated the timeline of developing endometrial carcinoma and its severity [46]. The deletion of *epithelial cadherin* (*Cdh1*) or the tumor suppressor *liver kinase B1* (Lkb1) along with *Pten* increases the severity of endometrial cancer invasiveness in mice [9,47]. The use of this genetic approach should facilitate the identification of gene targets for the treatment of *Pten*-related endometrial cancer. By example, the pharmacological inhibition of mammalian target of rapamycin (mTOR) signaling was shown to substantially inhibit the growth and progression of *Pten*/*Lkb1*-deficient endometrial cancer. Given that E2 enhances and P4 attenuates type 1 endometrial cancer, the *Pten* loss-of-function model of endometrial cancer has utility in understanding the endocrine actions of these female sex steroids [2,48,49,50].

PGRMC2 expression was not different between endometrial cancer tissue compared with endometrial tissue obtained during the proliferative and secretory phase of the menstrual cycle (Figure 1). The ablation of *Pgrmc2* reduced the percentage of *Pten^+/d^* mice developing hyperplasia by about 50% and those developing endometrial cancer by 66% (Table 2 and Figure 3). More striking was the survival data when both *Pten* alleles were ablated. Here, 100% of *Pten^d/d^* mice developed endometrial cancer and were euthanized prior to the end of the nine-month trial. In contrast, 100% of *Pten^d/d^; Pgrmc2^d/d^* mice survived to nine months despite all developing endometrial cancer suggesting the Pgrmc2 not only attenuates tumor growth, but also the likely metastatic spread.

The actions of PGRMC2 are consistent with prior studies in which *PGRMC1* knockdown in endometrial cancer cells dramatically reduced xenograft tumor growth in immunocompromised mice while also elevating chemosensitivity [31]. Similar findings were observed in PGRMC1-deplete ovarian and breast cancer xenograft tumors [30,32]. Our prior evaluation of *Pgrmc1^d/d^* and/or *Pgrmc2^d/d^* endometrial tissue demonstrated that the ESR1 and PGR levels do not differ from the corresponding control tissues in which *Pgrmc* genes remain intact [16,17]. Consistent with these findings, *Esr1* and *Pgr* mRNA levels did not change following ablation with *Pten* and/or *Pgrmc2* in the present study (Figure 5). Additional studies are needed to determine if *Pgrmc2* fits the generally accepted definition of a proto-oncogene, a normal gene that produces proteins which helps cells grow and proliferate, or to simply help cells survive. The ablation of *Pgrmc2* clearly attenuated the incidence/development, progression, and aggressiveness of *Pten* loss-of-function-induced endometrial cancer by inhibiting endometrial glandular epithelial cell proliferation (Figure 6). A great deal of excitement surrounds the notion that inflammation initiates neoplastic transformation and/or promotes the tumor progression of solid tumors [51,52]. The conditional ablation of *Pten* using *Pgr-Cre* mice elevates endometrial inflammation [6]. We assessed the recruitment of mast cells in *Pten^+/fl^; Pgrmc2^fl/fl^*, *Pten^+/d^*, and *Pten^+/d^; Pgrmc2^d/d^* mice and observed an increase in endometrial mast cells in *Pten^+/d^* mice compared with *Pten^+/fl^; Pgrmc2^fl/fl^* control mice (Figure 7). The ablation of *Pgrmc2* had no effect on mast cell recruitment, indicating that the ability of a *Pgrmc2* deficiency to slow the progression of endometrial cancer on a *Pten* haploinsufficient background does not stem from changes in mast cell recruitment or degranulation.

## 5. Conclusions

Through the use of a well-established mouse model of *Pten* loss-of-function-induced endometrioid endometrial carcinoma, this study demonstrates that the loss of *Pgrmc2* attenuates endometrial hyperplasia and cancer incidence and severity, in part, by inhibiting endometrial glandular epithelial cell proliferation. These findings on a member of the PGRMC family conducted using a mouse model of endometrial cancer advance our prior studies demonstrating that the knockdown of *PGRMC1* in a human endometrial xenograft tumor attenuated tumor development, growth, and progression in vivo [31], a finding also supported by ovarian and breast cancer xenograft studies [30,32].

## Figures and Tables

**Table 1:** Genotyping and qPCR primers.

Gene name	Primers
*Esr1*	F: CCAAAGCCTCGGGAATG R: CTTTCTCGTTACTGCTGG
*Pgr*	F: ATGGTCCTTGGAGGTCGTAA R: CACCATCAGGCTCATCC
*Pgrmc2* (genotyping)	F: ATGGTGGATCATAACCATCTG R: CCTTGATTTCTAAGTGAAAGC
*Pten* (genotyping)	F: CCAGCACTCTGCGAACTGAG R: AAGTTTTTGAAGGCAAGATGC
*Cre recombinase* (genotyping)	P1: ATGTTTAGCTGGCCCAAATG P2: TATACCGATCTCCCTGGACG P3: CCCAAAGAGACACCAGGAAG

**Table 2:** Endometrial hyperplasia and cancer incidence in *Pgr^Cre/+^*-driven *Pten* and/or *Pgrmc2* conditional mutant mice.

Genotype	n	Age (Months)	Normalized Tract Wet Weight * (g)	Hyperplasia (%)	Cancer (%)
*Pten^+/d^*	9	9	8.41 ± 1.74	88.9	22.2
*Pten^+/fl^*	13	9	3.71 ± 0.29	0	0
*Pten^+/d^Pgrmc2^d/d^*	28	9	7.09 ± 0.96	46.4	7.1
*Pten^+/fl^Pgrmc2^fl/fl^*	27	9	3.30 ± 0.19	0	0
*Pten^d/d^*	8	4–9	56.63 ± 20.29	100	100
*Pten^fl/fl^*	4	9	3.30 ± 0.32	0	0
*Pten^d/d^Pgrmc2^d/d^*	9	9	79.63 ± 29.81	100	100
*Pten^fl/fl^Pgrmc2^fl/fl^*	8	9	3.17 ± 0.27	0	0
*Pgrmc2^d/d^*	14	9	2.75 ± 0.56	0	0
*Pgrmc2^fl/fl^*	3	9	**	0	0

* wet weight: mass of total tract from ovariectomized female (uterus + cervix + vagina) (g)/body weight (g) × 1000. ** data not collected.

## Data Availability

The original contributions presented in this study are included in the article. Further inquiries can be directed to the corresponding author.
